# HCV incidence is associated with injecting partner age and HCV serostatus mixing in young adults who inject drugs in San Francisco

**DOI:** 10.1371/journal.pone.0226166

**Published:** 2019-12-10

**Authors:** Kimberly Page, Jennifer L. Evans, Judith A. Hahn, Peter Vickerman, Stephen Shiboski, Meghan D. Morris

**Affiliations:** 1 Department of Internal Medicine, University of New Mexico Health Sciences Center, Albuquerque, New Mexico, United States of America; 2 Department of Epidemiology and Biostatistics, University of California San Francisco, San Francisco, CA, United States of America; 3 Department of Medicine, University of California San Francisco, San Francisco, CA, United States of America; 4 Population Health Sciences, University of Bristol, Bristol, United Kingdom; University of New South Wales, AUSTRALIA

## Abstract

**Background:**

HCV incidence is increasing in the US, notably among younger people who inject drugs (PWID). In a cohort of young adult (age<30 years) PWID in San Francisco we examined whether ‘injecting partner mixing’ factors, i.e. age of partner and knowledge of their HCV serostatus, were associated with HCV transmission.

**Methods:**

In 448 susceptible PWID studied prospectively. All participants were asked to report characteristics and behaviors they engaged in with up to 3 *injecting partners* defined as “people whom you injected the most with” in the past month”. These partnerships did not specify that drugs or injecting equipment was shared. HCV incidence was estimated by age of up to 3 injecting partners, categorized as: (i) all <30; (ii) mixed-age (<&≥30); and (iii) all ≥30 years and perceived knowledge of the HCV status of participants’ injecting partners’ HCV status. Interaction was evaluated between partnership age categories and perceived HCV status of partners.

**Results:**

Between 2006–2018, overall HCV incidence (/100 person years observation [pyo]) was 19.4 (95% CI: 16.4, 22.9). Incidence was highest in those with mixed-age partnerships: 28.5 (95% CI: 21.8, 37.1) and those whose partners were all <30 (23.9; 95% CI: 18.8, 30.4), and lowest if partners were ≥30 (7.5; 95% CI: 4.8, 11.8). In a multivariable analyses adjusting for age, sex (of index), injection frequency, and injection partnership ‘monogamy’, we found evidence for an interaction: the highest HCV incidence was seen in PWID whose partners were all <30 and who knew at least one of their partners was HCV-positive (58.9, 95% CI: 43.3, 80.0; p<0.01).

**Conclusions:**

Younger injectors are more likely to acquire HCV from their similarly-aged peers, than older injecting partners. Protective seroadaptive behavior may contribute to reduce incidence. These findings can inform new HCV prevention approaches for young PWID needed to curb the HCV epidemic.

## Introduction

Hepatitis C virus (HCV) incidence is highest among younger, recent initiates to injection drug use in the United States (US) [1], and over the past decade infection has increased among younger people who inject drugs (PWID) in both urban and rural areas [2–4]. In studies that have prospectively studied HCV incidence, rates are markedly high; in a San Francisco cohort of young adult (age<30 years) PWID (UFO Study), HCV has hovered at 25/100 person-years observation (pyo) since 2002 [5, 6]. And, in rural Kentucky, incidence in PWID is estimated at 21.2/100 pyo (95% CI: 16.2, 27.7) (J. Havens, personal communication).

HCV infection is an important public health problem: over time, chronic infection leads to substantial liver disease, including cirrhosis and hepatocellular carcinoma, and HCV-associated mortality surpasses all other reportable infectious disease mortality in the US [7]. Temporally, in PWID, HCV infection almost always precedes HIV infection. For instance, reports of HCV foreshadowed the recent HIV outbreaks in Indiana [8, 9] and more recently in West Virginia and Massachusetts [10–13].

Multiple individual level exposures that increase risk for HCV are recognized including—the duration and frequency of injecting, sharing needles, syringes and injecting equipment, and female sex [5, 14–16]. Some researchers have suggested that younger injectors are more likely to acquire HCV infection from older injectors [17], especially young women [18], yet this has not been studied in quantitative analyses. The higher HCV prevalence observed in older PWID relative to younger PWID [19–21] could contribute to higher transmission rates from older to younger groups. PWID under age 35 have been shown to have higher injection related risk behavior relative to those older than 35 [20]. Medication for opioid use disorder and syringe service programs, which reduce the frequency and high risk of injecting and syringe sharing, have been shown to significantly reduce risk of HCV acquisition [22] including in young adult PWID [23]. But, social factors within injecting partnerships are also operative, amplifying both individual risk behaviors and subsequent infection risk [5, 24, 25]. Our group has studied injecting partnerships as an exposure variable in several epidemiological studies, defined broadly as “*people whom you injected* the most with- in the past month”, asked in order of first, second and third most frequent partners [5, 24, 25]. Injecting partners were not necessarily syringe sharing partners. The reasoning behind our use of the term “injecting partnerships” is that for a great majority of PWID, injecting drugs is a highly social activity not conducted in isolation [18, 26, 27]. Injecting partnerships are formed for various reasons, including for economic benefits, such as to share drugs and the associated costs [5, 28, 29], because of the risk environment (eg., safety), and for social resource and/or intimate and romantic reasons [30, 31]. Young adult PWID in overlapping injecting/sexual partnerships have five-fold higher odds of receptive needle sharing and more frequent injecting than PWID in injecting only partnerships [32]. In the UFO Study, both women and men who report injecting with sex partners relative to those who do not report these overlapping partnerships, have higher risk for HCV (adjusted Hazard Ratio (aHR) = 2.23; 95% CI, 1.9, 2.6) [24]. We also found that the smaller aged difference (< 5 years vs. ≥10 years) between injecting partners was associated five-fold higher odds of receptive needle sharing, a significant risk exposure for HCV [25]. Further, those that thought their partner was HCV-positive were significantly less likely to engage in receptive needle sharing with that partner, suggesting an avoidance of risk within injecting partnerships when HCV status was known, a potentially protective behavior. In this paper, due to accumulating HCV infections, we are able to follow up on these previous findings of behavioral outcomes higher injecting risk exposures found to be associated with age and lower risk behaviors associated with knowledge of partner’s status and examine associated HCV incidence outcomes. Because of these previous findings and the view of many that younger and more recent initiates have less knowledge about injecting safety and resources to reinforce risk reduction, we hypothesized that age-related injection partner mixing may be associated with HCV risk: young adult (<30 years old) injectors with younger injecting partners will have higher risk of HCV, and knowledge of partners’ serostatus may moderate this risk due to them taking necessary precautions. To test this we: (1) estimated incidence of HCV among young adult PWID by the age mix of their three most frequent injecting partners; and (2) examined effect modification by perceived knowledge of injecting partner’s HCV serostatus. The goal of this analyses is to better understand how age mixing and knowledge of injecting partners’ HCV status impacts HCV transmission and to inform prevention approaches to reduce acquisition and transmission of HCV among PWID.

## Methods

### Setting and population

The UFO Study is an ongoing prospective observational study of young adult (enrolled at <30 years old) active injectors, initiated in 2003 and ongoing to 2018. Details of eligibility, enrollment methods and follow up have been presented in detail in previous publications [5, 6]. In brief, young PWID are located and recruited using targeted street-based outreach in San Francisco neighborhoods where they were known to congregate and invited to a study field site for eligibility screening. Eligible participants self-reported injection drug use in the prior 30 days, being <30 years of age, were able to speak English, had no plans to travel outside of the San Francisco Bay Area for at least 3 months, and self-reported negative or unknown anti-HCV status, or if anti-HCV positive, then negative or unknown HCV RNA status. The Institutional Review Board (IRB) at the University of California reviewed and approved the protocol. All participants provided written informed consent, witnessed by study personnel. IRB determined that minors were emancipated.

### Data collection and prospective follow-up

Eligible consenting participants were asked to complete a baseline interviewer-administered structured questionnaire and provide blood samples for HCV testing. The questionnaire asks about participant socio-demographics, sexual risk behaviors/exposures, injecting exposures (e.g. frequency of injecting, number of people injected with, types of drugs injected), alcohol use (starting in 2006[33], and prevention and health service use. All participants who report *any* injecting partners are also asked to report on these partnerships; specifically, on up to three injecting partners with “*whom he/she had injected the most with in the prior month*”. This broad definition did not require that drugs or injecting equipment was shared. This broad definition aimed to elicit the most partnerships without being stigmatizing, potentially leading to under-reporting. The injection partnership questions include items on each injecting partner’s characteristics including: age, gender (male, female, transgender), nature of relationship (e.g., romantic/sexual, family, friend), perception of partner’s HCV serostatus, and the basis of that knowledge (e.g., was told by partner, was told by someone else, saw HCV test results, “just know”). Within each partnership they are asked to report: frequency of injecting together, preparing drugs together in shared equipment, and sharing injecting equipment, including syringes, cookers and cottons. All participants are asked to return for quarterly visits for ongoing survey and serological data collection. This analysis uses questions from the survey on exposures overall, and from the injecting partnerships-specific questions (including data collected at baseline and quarterly interviews). Participants who reported who reported ≥1 injecting partner at the seroconversion or censoring visit were included.

Participants were tested for HCV antibodies (anti-HCV) and HCV ribonucleic acid (RNA). Prior to 2012 all participants underwent phlebotomy for anti-HCV (using standard laboratory-based anti-HCV), and for a qualitative HCV RNA status determination using a nucleic acid amplification test (Procliex HIV-1/HCV assay, Gen-Probe Inc., San Diego, CA, and marketed by Novartis Vaccines & Diagnostics, Emeryville). Beginning in May 2012 anti-HCV testing was primarily conducted using a rapid test (OraSure© Technologies: Bethlehem, PA).

### Analyses

Descriptive statistics (measures of central tendency (means and medians) and statistical dispersion (standard deviation (SD) and interquartile range (IQR)) were tabulated on baseline data of participants who reported at least one injection partner. Incidence was estimated at the individual level using time-to-event analyses with infection estimated based on three exposure categories of the age of participants’ reported injecting partners as follows: (1) all partners <30 years; (2) all partners ≥30 years; or (3) mixed age partnerships (under and over 30 years). This cut-off was chosen since all participants are initially enrolled under age 30 years. Study participants also reported their perception (referred to as ‘knowledge’) of their injecting partners’ HCV serostatus, which was categorized as: (1) any HCV-positive partner; (2) all HCV-negative partners; or (3) mixed HCV-negative and unknown status partners. Cumulative person-time HCV incidence rates by injecting partners age group category and knowledge of partners’ HCV status category were calculated and 95% confidence intervals (95% CI) for the rates assuming a Poisson distribution. Injecting partner age category and partners’ HCV status category were obtained from data reported at the interview of the first HCV-positive blood sampling for those with new infections identified, and at the time of the last HCV-negative blood sampling for those with no incident HCV during follow up. HCV infection dates were imputed as the midpoint of the interval between the dates of the last observed HCV-negative test result and either the first HCV RNA-positive or first anti-HCV positive test result. For 63 of 140 incident infections, HCV RNA was detected in the acute window prior to antibody seroconversion. For these cases, the date of infection was estimated as the date 30 days prior to the first positive HCV RNA test result. This date is used because the period in which HCV RNA is detectable but anti-HCV is not detectable is, on average, 51 days [34]. Time at risk was defined as time from study enrollment to date of HCV infection. Subjects who were HCV susceptible (anti-HCV and RNA negative) were entered into the analysis at the baseline visit and remained until date of new HCV infection, or were censored at August 1, 2018, or last interview/visit date. Censoring at last visit could occur for various reasons, including loss to follow-up and death. Bivariable Cox proportional hazards models were fit to evaluate the association between selected partnership characteristics including mix of partners by age, and mix of partners by HCV status, as well as the number of partners (injection and sex partners), number of male partners, number of female partners, number of opposite sex partners, number of partners aged 5-years older, number of partners under age 30, receptive syringe sharing, and ancillary equipment sharing. Multivariable Cox proportional hazards models were fit to evaluate the association between injecting partner age category and knowledge of HCV status on HCV incidence, adjusting for sex and age of index, frequency of injecting, sharing syringes or ancillary equipment, and injecting monogamy (defined as having one vs. multiple injection partners). Except for sex, all variables entered into the model were obtained from interviews conducted when HCV was first detected (which assessed prior 30 day or 3-month exposures), or among negatives, the last HCV-negative test date. Interaction was evaluated using a product term of partner age category and partner HCV status. Cox models were checked for violation of the proportional hazards assumption by assessing scaled Schoenfeld residuals and log-minus-log survival plots for patterns of non-proportionality. All analyses were conducted with Stata (version 15, College Station, TX).

## Results

Between April 2003 and August 2018, a total of 2,486 people was screened, 930 (37.4%) met eligibility criteria and completed a baseline interview, of whom 710 (76%) were HCV negative (RNA and/or anti-HCV), and 522 (74%) had at least one follow-up interview. A total of 448 (85.8% of 522) participants reported on at least one to three injecting partnerships each, for a total of 986 injecting partnerships is included in this analysis. Of the 710 completing baseline interviews, those reporting having at least one partnership compared to those reporting *no* partnerships were significantly more likely to be female (31% vs. 21%, p = 0.04), inject heroin or heroin mixed with other drugs ‘*most days*’ (vs. methamphetamine or other drugs; (67% vs. 55%, p = 0.02), inject every day in the past month (38% vs. 16%, p<0.01), and attend syringe service programs (80% vs. 59%, p<0.01). Participants who remained in follow up (N = 522) vs. those who did not (N = 188), respectively, were older (median age = 24.0 vs. 23.1, p = <0.01), and more likely to have ever been tested for HCV (71.2% vs. 56.6%, p<0.01) and HIV (85.5% vs. 78.5%, p = 0.03), to attend syringe service programs (79.3% vs. 69.6%, p<0.01), to have received mental health services in the past 3 months (24.5% vs. 11.0%, p<0.01), to inject every day in the past month (38.1% vs. 24.1%; p<0.01), to use non-injected methamphetamine (64.8% vs. 54.3%, p = 0.01); and less likely have a AUDIT-C score indicative of probable alcohol dependence (12.5% vs. 24.4%, p<0.01).

Baseline characteristics of the sample at risk are shown in Table 1. Median age was 24.0 (IQR: 21.4, 26.4), just under one-third (29.2%) were women, 32.6% did not graduate high school. In the prior 3 months, 74.6% reported they were homeless or unstably housed, and 25.6% had been incarcerated. At baseline, prior to testing, 37.8% of participants did not know or had never been previously tested for HCV. The study sample reported a median 3.8 years of injecting (IQR: 1.7, 7.2). In the past month, median number of injecting partners reported was 4 (IQR 2, 10), 38.2% were daily injectors, and in the past 3 months: 81.5% reported injecting heroin or heroin mixed with other drugs, 30.3% reported receptive needle sharing, 61.7% shared ancillary equipment, and 44.1% reported injecting with 5 or more people. Median duration of follow-up in the analysis population was 10 months (IQR: 3.0–29.0 months).

**Table 1:** Baseline demographic characteristics and recent injecting behaviors of hepatitis C virus (HCV) negative young injection drug users in San Francisco with ≥1 injecting partner (N = 448).

Demographic characteristics	N or Median	% or Interquartile Range (IQR)
Age in years (median; IQR)	24.0	21.4, 26.4
Female	131	29.2
Less than high school education	145	32.6
White	294	65.6
Homeless/marginally housed, prior 3 months	334	74.6
Ever incarcerated (jail or prison)	359	80.1
Incarcerated (jail or prison), prior 3 months	114	25.6
Traveled outside of San Francisco, prior 3 months	188	42.0
**Participant HCV/HIV status**		
Self-reported HCV status (n = 386)*		
Negative	234	60.6
Positive	4.0	1.0
Unknown	31	8.0
Never tested	115	29.8
Self-reported HIV status (n = 445)		
Negative	319	71.7
Positive	10	2.2
Unknown	22	4.9
Never tested	69	15.5
**Participant drug and alcohol use**		
Audit-C score, prior month (n = 360)^#^		
Low risk	175	48.6
Hazardous	140	38.9
Probable dependent	45	12.5
Drank alcohol 21 or more days, prior month	93	20.8
Smoked crack cocaine, prior 3 months	247	55.3
Snorted or smoked methamphetamine, prior 3 months	295	65.9
Years since first injected, median (IQR)	3.8	1.7, 7.2
Number of days injected, prior 30 days, median (IQR)	25	10, 30
Injected every day, prior 30 days	171	38.2
Number of people with whom participant reported having injected with in the prior month; median (IQR)	4	2, 10
**Participant recent injecting exposures: prior 3 months**		
Injected heroin (by itself or mixed with other drugs)	365	81.5
Injected methamphetamine	272	60.7
Injected powder cocaine	112	25.0
Injected crack cocaine	319	78.4
Obtained needles/syringes at syringe service program	349	78.1
Injected with the same needle/syringe >1 time	425	95.1
Any receptive needle sharing (yes)	135	30.3
Any shared ancillary equipment (yes)	276	61.7

* Participants who knew they were HCV viremic (RNA-positive) were screened out at baseline

# Sample size is lower as Audit C measures were added in 2006

HCV incidence (per 100 pyo) was higher in those whose injecting partners were all <30 years of age: 23.9 (95% CI: 18.8, 30.4), and in those with mixed-age injecting partnerships (28.5; 95% CI: 21.8, 37.1) compared those whose partners were ≥30 years of age (7.5; 95% CI: 4.8, 11.8). Among those who did not report any injecting partnerships, and who were excluded from the analysis, we observed 15 seroconversions an incidence rate of 16.2 (95%CI: 9.7, 26.8) per person year of observation.

Characteristics of the injecting partnerships and associations with HCV incidence are shown in Table 2. Over half (52.2%) of participants reported on three injecting partners: 44.9% reported that all of their injecting partners were <30 years old, and 38.6% that all of their injecting partners were HCV-negative. Most had at least one male partner (88.1%), and 37.0% had one or more injecting partners who were also sex partners. Less than a third (32%) of participants in this sample reported having only one injection partner. Knowledge of partners’ HCV status differed significantly by this indicator of monogamous injecting relationship: those with only one partner compared to those with multiple partners were significantly less likely to have a known HCV-positive partner (19.4% vs. 44.7%), or HCV-status unknown partners (30.9% vs. 41.8%) and more likely to inject only with HCV-negative partners (49.6% vs. 13.6%). In bivariable Cox regression analyses, compared to those whose partners were ≥30 years, HCV acquisition was associated with having all injecting partners <30 years (HR = 2.5: 95% CI: 1.5, 4.2) or a mix of partners both <&≥30 years (HR = 2.9: 95% CI: 1.7, 5.0). Compared to those whose partners were all HCV-negative, HCV acquisition was associated with having a mix of HCV-negative and HCV-unknown partners (HR = 2.0: 95% CI 1.2, 3.5) and having any HCV-positive partner (HR = 4.1: 95% CI: 2.4, 6.8). HCV acquisition was significantly associated with having ≥3 injection partners (vs. 1; HR = 3.6, 95% CI: 2.3, 5.6), having ≥2 male injecting partners (vs. 1; HR = 3.5, 95% CI: 1.8, 7.1), and reporting multiple partners of the opposite sex (HR: 2.0, 95CI: 1.3, 3.1) relative to no partners of the opposite sex. Receptive needle sharing and sharing ancillary equipment were both significantly associated with incident HCV in bivariate analyses. However HCV was not associated with having injecting partners who were also sex partners (Table 2), and not associated with the index partner’s age (HR: 1.0, 95% CI: 0.9, 1.0) or sex (HR: 0.8, 95% CI: 0.6, 1.2).

**Table 2:** Injecting partnership characteristics* and bivariate associations with HCV incidence.

Characteristic	N	%	HR	95% CI
Injecting partner age mix				
All ≥ 30 years	96	21.4	1.0	
Mixed over & under 30 years	151	33.7	2.9	1.7, 5.0
All < 30 years	201	44.9	2.5	1.5, 4.2
HCV serostatus^#^ of injecting partners				
All HCV negative	173	38.6	1.0	
Mix of HCV negative & unknown	111	24.8	2.0	1.2, 3.5
Any HCV positive	164	36.6	4.1	2.4, 6.8
Number of injecting partners				
1	139	32.0	1.0	
2	70	15.6	1.6	0.9, 3.0
3	239	52.4	3.6	2.3, 5.6
Number of female injecting partners				
0	236	52.7	1.0	
1	162	36.2	1.3	0.9, 1.9
2+	50	11.2	1.1	0.6, 2.0
Number of male injecting partners				
0	53	11.8	1.0	
1	174	38.8	1.7	0.8, 3.5
2+	221	49.3	3.5	1.8, 7.1
Number of opposite sex injecting partners				
0	157	350	1.0	
1	171	38.2	1.2	0.8, 1.9
2+	120	26.8	2.0	1.3, 3.1
Number of partners who are also sex partners				
0	282	63.0	1.0	
1	147	32.8	1.3	0.9, 1.8
2+	19	4.2	0.8	0.3, 2.1
Number of partners who are 5 years older				
0	189	42.2	1.0	
1	170	38.0	0.7	0.5, 1.1
2+	89	19.9	1.0	0.7, 1.6
Any receptive needle sharing (yes)	94	22.1	1.4	1.0, 2.1
Any shared ancillary equipment (yes)	297	70.4	3.4	2.1, 5.6

*The three injecting partners with whom he/she “*had injected with the most in the past month*”; measured at censoring visit. Partnership characteristics were obtained from interviews conducted when HCV was first detected (which assessed prior 30 day or 3-month exposures), or among negatives, the last HCV-negative test date).

# Injecting partner serostatus as known/perceived by respondent.

**Fig 1:**
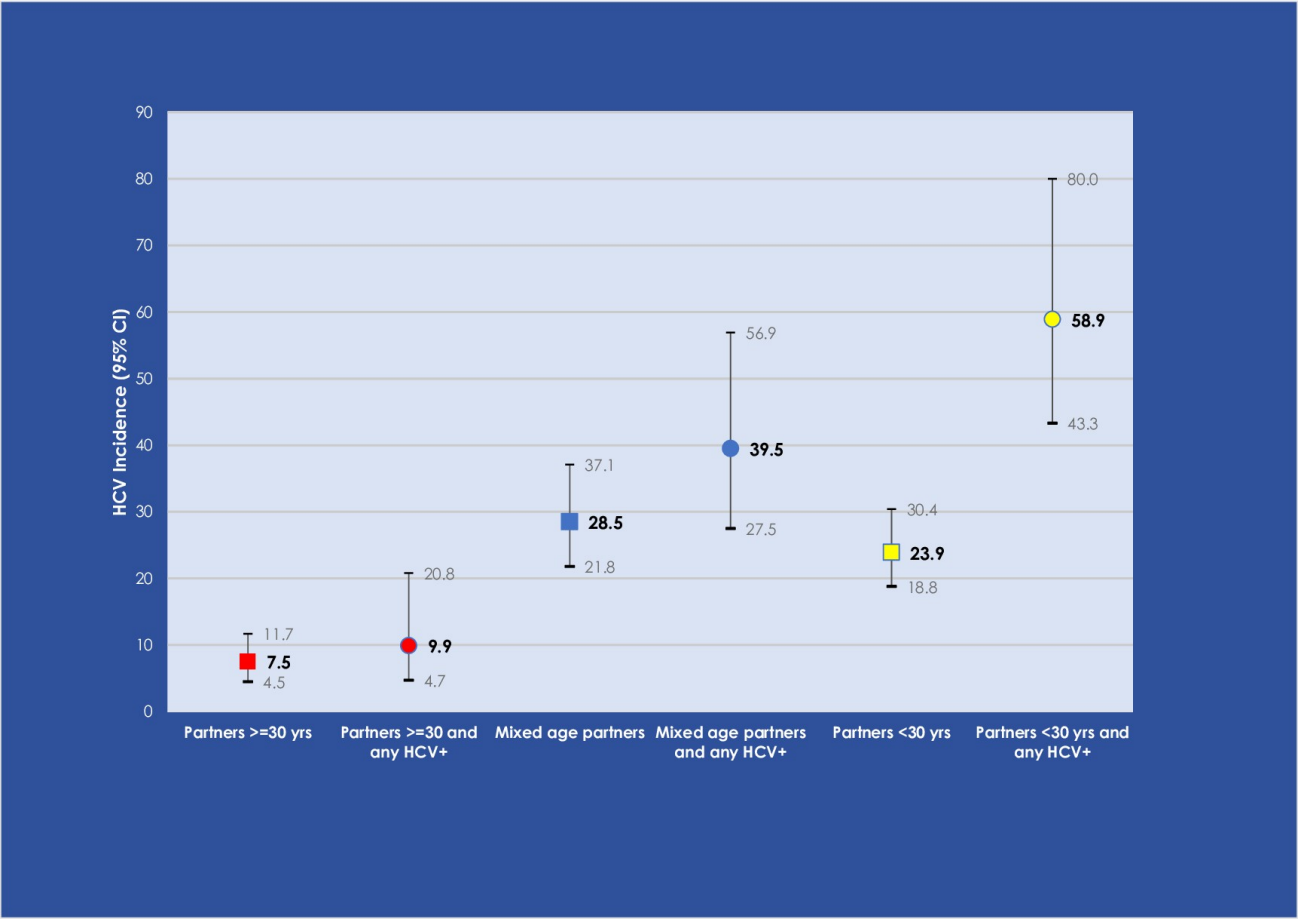
HCV incidence per 100/person years observation (and 95% Confidence Interval) among young people who inject drugs by injecting age and HCV serostatus partner group.

Multivariable analyses adjusting for sex and age of respondent, frequency of injecting, and number of partnerships are shown in Table 3. HCV acquisition was independently associated with having all injecting partners <30 years, knowledge that at least one partner was HCV-positive (adjusted HR (aHR) = 2.6, 95% CI: 1.3, 5.3), and with having mixed age partnerships (<&≥30 years) and knowledge that at least one partner was HCV-positive (aHR = 2.1, 95% CI: 1.0, 4.3). A significant interaction was found between partnership age mix and perceived knowledge of partners HCV status (p = 0.03). Fig 1 shows incidence rates by injecting age mix and knowledge of HCV serostatus group: the highest HCV incidence was in those whose injecting partners were <30 years of age and any partner was known to be HCV-positive, over five-fold higher than observed in those whose partners were all ≥30 years and any partner was known to be HCV-positive (Incidence Rate Ratio (IRR) = 5.9 (95% CI: 2.7, 13.2)).

**Table 3:** Multivariable models of HCV incidence by injection partner mixing: Age and HCV serostatus and select characteristics in young adult PWID; n = 448*.

	Adjusted HR	95% CI
Partner age and HCV serostatus mix#		
All ≥30 years & mix of HCV-negative & -unknown	1.0	
All ≥30 years & any HCV-positive	1.7	0.7, 4.4
Mixed over & under 30 years and mix of HCV-negative and–unknown	1.4	0.7, 3.0
Mixed over & under 30 years & any HCV-positive	2.1	1.0, 4.3
All <30 & mix of HCV-negative & unknown	1.2	0.6, 2.5
All <30 & any HCV-positive	2.6	1.3, 5.3
Respondent is male	0.8	0.6, 1.2
Injected every day of past month	2.7	1.9, 3.9
Age of respondent	0.9	0.85, 0.95
Injects exclusively with one partner	0.6	0.3, 1.0

*Measured at censoring

# Interaction of age and HCV serostatus mix p-value = 0.03

## Discussion

In this study of HCV incidence in young injectors, rates of infection differed by injecting partner age and knowledge of HCV status of these partners. Those at lowest risk for HCV were those whose injecting partners were all older—over 30 years of age, including if they knew that at least one of these was HCV-positive. Although researchers have suggested that younger injectors are more likely to get HCV infection from older injectors, studies have not directly assessed this [17, 18]. Our study provides the first evidence that younger injectors injecting with persons of similar age are more likely to become infected with HCV, independent of other exposures including sex, injection frequency and equipment sharing. In a study of HCV phylogenetic clustering among PWID in Vancouver, Canada, younger age, HIV co-infection, HCV seroconversion and recent syringe borrowing were independently associated with membership in an HCV cluster [35], and in further examination of infections in pairs of clusters, researchers showed that viruses from 28 younger (<27 years) PWID were more likely to be closely related to viruses from other younger injectors, with fewer such events in older and younger injection pairs [36]. Examining the intersectional nature of social relationships for which injection drug use occurs within can substantially inform disease transmission patterns and inform control and prevention methods. Examining partner mixing is one approach to examine these risks and disease patterns [37]. Older adults have been shown to mix with their and all other age groups but younger adults do not–instead mixing more with younger adults [38, 39]. Collectively, these results extend our previous findings of higher exposure risk within pairs of younger PWID [25] and suggests that young PWID are at highest risk for forward transmission.

Risk of HCV acquisition is further impacted by perceived HCV status of injecting partners. Participants with younger injecting partners who also reported that some of those partners were HCV positive had over five-fold higher incidence of HCV relative to those in injecting relationships with older partners that included some partners with HCV infection. In previous analyses, young adult PWID had lower odds of reporting injection equipment sharing with HCV-positive partners relative to older PWID [25]. These updated data are consistent with those previously published; the proportion of respondents who report any receptive syringe sharing (22%) and any sharing ancillary equipment (70.4%) within injecting partnerships was similar to those previously published (23% and 64%, respectively). Our results suggest that protective seroadaptive behavior may vary with age. Younger injectors, notably those with shorter injecting careers acquire HCV very rapidly [1, 21], in association with higher risk injecting exposures [40]. We have previously reported that young adult PWID did not report reducing lending of previously used injecting equipment to others or ancillary equipment sharing after having been informed of their HCV infection following testing [41]. It is possible that the older partners are the ones who are engaged in more protective behaviors, resulting in protection for younger PWID. A study in San Diego found that accurate knowledge of HCV infection increased with age [42]. It is possible that younger injectors with older partners may be more likely to be injecting partner monogamous, thus limiting exposure to other HCV infected persons. Since HCV prevalence is higher in older injectors, these results suggest that behavioral factors, potentially seroadaptive behaviors, may be operational in reducing HCV incidence, as has been seen with HIV in MSM dyads [43]. Interpersonal dynamics that contribute toward how partners inject is associated with trust, intimacy and cooperation, which also contribute to perceived risk for infection [44]. These injecting related interpersonal dynamics may differ based on age differences in injecting partnerships. Further exploration to identify who and how these seroadaptive mechanisms can be harnessed for greater dissemination, potentially from older-younger to young-younger groups or in injecting dyads. Conversely, awareness of partner's HCV status may not influence injection decisions if HCV not considered a serious concern, or if HCV is thought to be unavoidable [45, 46]. There may be age differences in the perception of HCV infection as an expected consequence of injecting and how this is dealt with in partnerships [47]. All of these factors may also have contributed to the lack of association found between sexual partnerships and HCV infection outcomes, a result we found to be unusual given previous research shows that overlapping sexual/injecting partnerships have been shown to be associated with differential risk exposures for HCV infection [24, 31, 48]. The interpersonal dynamics influencing behaviors sexual relationships may be different within injecting partnerships [44]. We note that even when the partners are not sexual partners, there may be close and reciprocating social ties formed through the relational qualities of injecting together not captured by the term ‘sex partner’ that is likely to be associated with a heightened risk of unsafe injection practices. In a cohort of PWID in Baltimore, Smith et al, 2018 [49] examined the propensity of mixing patterns and found that age assortative patterns were more likely in association with sexual mixing compared to drug-sharing contacts and casual contacts. The lack of association between sexual partnerships reported may be due to collinearity with age mixing, and other factors that drive partner selection, such as limited pools of partners, housing or non-housing patterns, the length of time partners have known and have injected with each other, or the additional roles played by some partners who may supply drugs, or for newer initiates, assist them with injection. As our findings also show, risk can vary with knowledge of serostatus and it is possible that “riskier” behaviors occur in sex partnerships that are seroconcordant. As HCV testing and awareness increase, negotiated safety may also change.

In addition to behavioral and interpersonal factors, HCV infection dynamics may contribute to higher incidence within younger injecting partnerships [50]. The acute infection phase may contribute to increased incidence due to the long seronegative viremic window (approximately two months) [34], and high viremia during this phase of infection [51]. Since HCV screening is primarily conducted using antibody testing, those with newly acquired infection, (i.e. younger PWID), which is also mostly asymptomatic, do not know they are infected. If they are in a phase of high-risk injecting behavior, lack of knowledge of active infection could contribute to further transmission. Testing all PWID for HCV RNA has potential to better inform people about their risk and contribute to behavior change. Prompt HCV RNA diagnosis could contribute to counselling for risk reduction and disclosure within partnerships as well as discussions about HCV treatment. Modeling studies have suggested that HCV treatment for PWID [52], and their partners [53] may lead to substantial reductions in HCV prevalence and transmission. Targeting treatment to younger PWID in ‘treatment as prevention’ approaches—those at highest risk has potential to be efficient and have high impact prevention results–- similar to what has been shown in models of HCV vaccination strategies [54].

This study has some potential limitations. Participants may misreport partnership characteristics in association with recall or social desirability bias. However, as risk would tend to be underreported, this would bias results toward the null. It should be noted that the addition of perceived HCV status to the model of HCV incidence results in large confidence intervals showing that this measure is unstable. These findings may not necessarily be generalizable to cohorts and networks of persons who inject drugs who live in other communities at risk. In Vietnam older injectors (30–39 years old) had higher injection equipment sharing patterns compared to other groups [49]. Social mixing, including injecting relationships may differ in association with race/ethnicity or other factors not seen in this primarily white cohort in San Francisco [55]. We did not measure duration, location or contact frequency within these injecting partnerships, but determined whether the relationship involved injecting. We also did not specifically restrict our analyses to age mixing within equipment sharing partnerships, and these results do not denote a transmission probability. We note that the including partnerships that may potentially have excluded equipment sharing results in a bias toward the null in terms of risk of HCV incidence. We further note we queried participants about the three partners with whom he/she had injected the most or with whom they spent the most time in the prior month, but some may have had more than three partners. Strengths of the study include repeated and accurate ascertainment of HCV incidence, including antibody and RNA testing.

In summary, findings from this study have important potential to inform new interventional approaches for HCV prevention in young PWID, which is critical given the expanding HCV epidemic. In addition to expanding testing to identify and promote treatment of HCV infection among PWID, promoting knowledge of protective behaviors, could be disseminated by older PWID. Note that with status quo in San Francisco, where there are significant programmatic prevention efforts aimed at PWID [56], HCV incidence has been stable and high in this young adult cohort. Younger people are the most challenging to retain in care and prevention, including treatment for opioid use disorder OUD [57, 58], using medications are more likely to be retained in care than those who receive only behavioral intervention [59]. Effective OUD treatment can substantively reduce HCV incidence [22], making the to test interventions that promote young people’s retention in these programs a priority. Given the widespread increases in HCV nationally in young adult PWID, new prevention interventions aimed at this younger population that address the social and cultural contexts of injecting partnerships should be tested.
